# Functional analysis and the importance of ligament reconstruction after soft tissue sarcoma surgery around the knee joint -a Tokai musculoskeletal oncology consortium study

**DOI:** 10.1186/s12957-025-03891-2

**Published:** 2025-06-13

**Authors:** Hiroshi Koike, Kunihiro Ikuta, Akihito Nagano, Hisaki Aiba, Kunihiro Asanuma, Shunsuke Hamada, Eiji Kozawa, Yuya Izubuchi, Katsuhisa Kawanami, Shiro Imagama, Yoshihiro Nishida

**Affiliations:** 1 Department of Orthopaedic Surgery, Nagoya University Graduate School of Medicine, Tsurumai, Showa, Nagoya, 466-8550 Aichi Japan; 2 Department of Orthopaedic Surgery, School of Medicine, Gifu University, 1-1, Yanagido, Gifu,, Gifu, Japan; 3 Department of Orthopedic Surgery, Nagoya City University, 1, Kawasumi Mizuho-cho, Mizuho-ku, Nagoya, Aichi Japan; 4 Department of Orthopaedic surgery, Mie University Graduate School of Medicine, 2-174, Edobashi, Tsu, Mie, Japan; 5 Department of Orthopaedic surgery, Aichi Cancer Center Hospital, 1-1, Kanokoden, Chikusa, Nagoya, Aichi Japan; 6 Department of Orthopaedic surgery, Nagoya Memorial Hospital, 4-305, Hirabari, Tenpaku, Nagoya, Aichi Japan; 7 Department of Orthopaedics and Rehabilitation Medicine, Faculty of Medical Sciences, University of Fukui, 23-3, Matsuokasimoaiduki, Eiheiji, Yoshida, Fukui, Japan; 8 Department of Orthopaedic surgery, Aichi Medical University School of Medicine, 1-1, Yazakokarimata, Nagakute, Aichi Japan; 9 Department of Rehabilitation Medicine, Nagoya University Hospital, 65, Tsurumai, Showa, Nagoya, Aichi Japan

**Keywords:** Soft tissue sarcoma, Knee joint, Ligament reconstruction, Functional outcome

## Abstract

**Background:**

Surgery for soft tissue sarcoma (STS) around the knee joint often requires extensive tissue resection, leading to significant functional impairment. This study aimed to evaluate knee function following surgery for STS around the knee joint and to assess the importance of ligament reconstruction in cases where the collateral ligaments were excised.

**Methods:**

We included 63 patients treated surgically at eight hospitals participating in the Tokai Musculoskeletal Oncology Consortium between 2008 and 2019. Tumor development within 3 cm proximal to the superior patella and 3 cm distal to the tibial articular surface was defined as the area surrounding the knee joint. The median patient age was 61 years (25 males; median follow-up period, 62 months). The median tumor size was 60 mm. The histological types included 18 undifferentiated pleomorphic sarcomas (UPS), 17 myxoid liposarcomas, 7 myxofibrosarcomas (MFS), and 21 others. Knee function was assessed using the Lysholm score.

**Results:**

The 5-year overall survival rate was 91%. Factors significantly associated with lower Lysholm scores were female sex (*p* = 0.01), tumor histology such as MFS and UPS (*p* = 0.01), adductor muscle resection (*p* < 0.001), lateral collateral ligament (LCL) resection (*p* = 0.002), patellar tendon resection (*p* = 0.019), and medial collateral ligament (MCL) resection (*p* = 0.024). The group with ≥ 4 resected muscles had significantly lower Lysholm scores than the group with < 4 resected muscles (*p* = 0.003). Multivariate analysis showed that the factors significantly associated with lower Lysholm scores were adductor muscle resection, female sex, and tumor histology (MFS and UPS). Among patients who required excision of the LCL or MCL, those who underwent ligament reconstruction had better functional scores than those who did not (*p* = 0.06).

**Conclusions:**

This study identified factors that significantly affected post-resection function in patients with STS of the knee joint. These findings may assist in predicting postoperative function after STS surgery around the knee joint, suggesting that ligament reconstruction may give a suggestive benefit for improving the function in cases requiring resection of the LCL or MCL.

## Highlights

- This study evaluated knee function after soft tissue sarcoma surgery around the knee joint.
- Factors affecting function included sex, tumor histology, and specific muscle/ligament resection.
- Ligament reconstruction improved outcomes, highlighting its potential role in enhancing post-surgical knee function.

## Background

Soft tissue sarcoma (STS) comprises various histological types, each of which dictates the choice of treatment, which include radiation therapy, chemotherapy, and surgery. However, the primary treatment modality is wide surgical excision of the tumor with the surrounding normal tissues [1]. This often results in extensive defects requiring reconstructive plastic surgery techniques such as myocutaneous flaps and skin grafts [2]. Following excision of the STS in the limbs, muscle and tendon deficits can occur, leading to functional impairment. Depending on the type of tissue removed, limb function can be significantly affected, necessitating functional reconstruction including tendon or muscle transfers and grafts [3–7].

The knee joint consists of the femur, tibia, and patella, along with other components such as joints and ligaments, and is supported by various muscles that maintain its function and stability. The knee joint consists of the medial, lateral, patellofemoral, and proximal tibiofibular joints. Several ligaments provide passive stability in all knee joint directions. In daily activities, the knee carries a large portion of the body weight, allowing a wide range of motion for flexion–extension and internal–external rotation [8].

The extent of tissue removal and the resultant functional impairments following STS excision, as well as the degree of functional reconstruction required, have not yet been fully clarified. While ligament reconstruction is anticipated to be beneficial in cases involving the excision of the collateral ligaments, the decision to reconstruct is clinically challenging. Reconstructive surgery extends the duration of surgery, increases the risk of infection, and may compromise other healthy tissues. Thus, determining the appropriateness of reconstruction is challenging with limited precedent reported in the literature. We, therefore, aimed to evaluate knee function following surgery for STS around the knee joint and to assess the importance of ligament reconstruction in cases where the collateral ligaments were excised.

## Methods

### Ethical approval

This study was approved by the Clinical Research Ethics Review Committee of Nagoya University Hospital (2019-029817509) and was conducted in accordance with the Declaration of Helsinki. Independent questionnaires administered to patients of eight hospitals of the Tokai Musculoskeletal Oncology Consortium were retrospectively reviewed.

### Patients

STS around the knee joint was defined as a tumor identifiable on contrast-enhanced MRI and located within 3 cm proximal to the superior pole of the patella and 3 cm distal to the joint level of the knee. The inclusion criteria were as follows: (1) patients with primary non-small round cell STS diagnosed between January 2008 and December 2019 and treated with wide resection; (2) patients who were followed up for at least 6 months after surgery and for whom various clinical information could be collected. We excluded patients who underwent amputation; those with large bone defects requiring osseous reconstruction; and pediatric patients under 14 years of age.

Perioperative chemotherapy and radiotherapy were administered according to the standards of each institution.

### Functional evaluation

The function of the affected limb was assessed using the Lysholm score, which is specifically designed to evaluate knee joint function after ligament injuries [9]. Functional scores and their association with various clinical factors and muscle resection were analyzed in 58 patients, excluding those with distant metastases at initial diagnosis and those who experienced recurrence.

### Statistical analysis

Survival was evaluated using Kaplan–Meier survival curves. Comparisons between two groups were conducted using Student’s t-test, whereas multivariate analysis was performed using multiple regression analysis. The correlation between the number of resected muscles and the Lysholm score was analyzed using Pearson’s correlation coefficient. Statistical analyses were conducted using statistical database software (IBM SPSS Statistics software for Microsoft Windows, Armonk, New York, US). Statistical significance was set at *P* < 0.05.

## Results

Of 77 cases identified based on the inclusion and exclusion criteria, we excluded 14 cases and those with insufficient information. Table 1 presents the patient demographics. The cohort comprised 25 males and 38 females, with a median age of 61 years (range 14–88 years) and a median follow-up duration of 62 months (range 10–133 months). All participants in this study were of Japanese ethnicity. The median tumor size was 60 mm. Regarding tumor depth, 35 cases were deep-seated, and 28 were superficial. High-grade malignancy was observed in 51 patients, whereas 12 patients had low malignancy. The histological types included 18 cases of undifferentiated pleomorphic sarcoma (UPS), 17 of myxoid liposarcoma, 7 of myxofibrosarcoma (MFS), 5 of synovial sarcoma, 4 of malignant peripheral nerve sheath tumor (MPNST), 3 of leiomyosarcoma, 3 of extraskeletal myxoid chondrosarcoma, and 6 others.

**Table 1:** Patient demographics (*n* = 63)

Variable	
Sex	
Male	25
Female	38
Age (y), (median, range)	61 (14–88)
Race/Ethnicity	Japanese (100%)
Follow up (M), (median, range)	62 (10–146)
Tumor Size (median, range)	60 mm (8–240 mm)
Lymph node metastasis	0
Distant metastasis	1
Tumor depth	
Deep	35
Superficial	28
Tumor grade	
High grade	51
Low grade	12
Unplanned excision	5
Histology	
Undifferentiated pleomorphic sarcoma	18
Myxoid liposarcoma	17
Myxofibrosarcoma	7
Synovial sarcoma	5
MPNST	4
Leiomyosarcoma	3
Extraskeletal myxoid chondrosarcoma	3
Others	6
Number of resected muscle (median, range)	2 (0–8)
Chemotherapy	
Neoadjuvant	5
Adjuvant	10
Neoadjuvant and Adjuvant	8
None	40
Radiotherapy	
Preoperative	2
Postoperative	2
Preoperative and Postoperative	1
None	58
Surgical margin	
R1	4
R0	59
Plastic reconstruction	
Pedicled flap	
Gastrocnemius	21
Free flap	
Latissimus dorsi	8
Anterolateral thigh	6
Local flap	2

Chemotherapy was administered as follows: neoadjuvant in 5 cases, adjuvant in 10, and both neoadjuvant and adjuvant in 8 cases. Radiotherapy was administered preoperatively in 2 cases, postoperatively in 2 cases, and both pre- and postoperatively in 1 case. The surgical margins were R0 in 59 cases and R1 in 4 cases. Plastic reconstructive procedures were performed using the gastrocnemius muscle in 21 cases, free latissimus dorsi muscle in 8 cases, free anterolateral thigh flap in 6 cases, and local flaps in 2 cases. During follow-up, local recurrence and distant metastasis were observed in 4 and 12 patients, respectively.

**Fig. 1:**
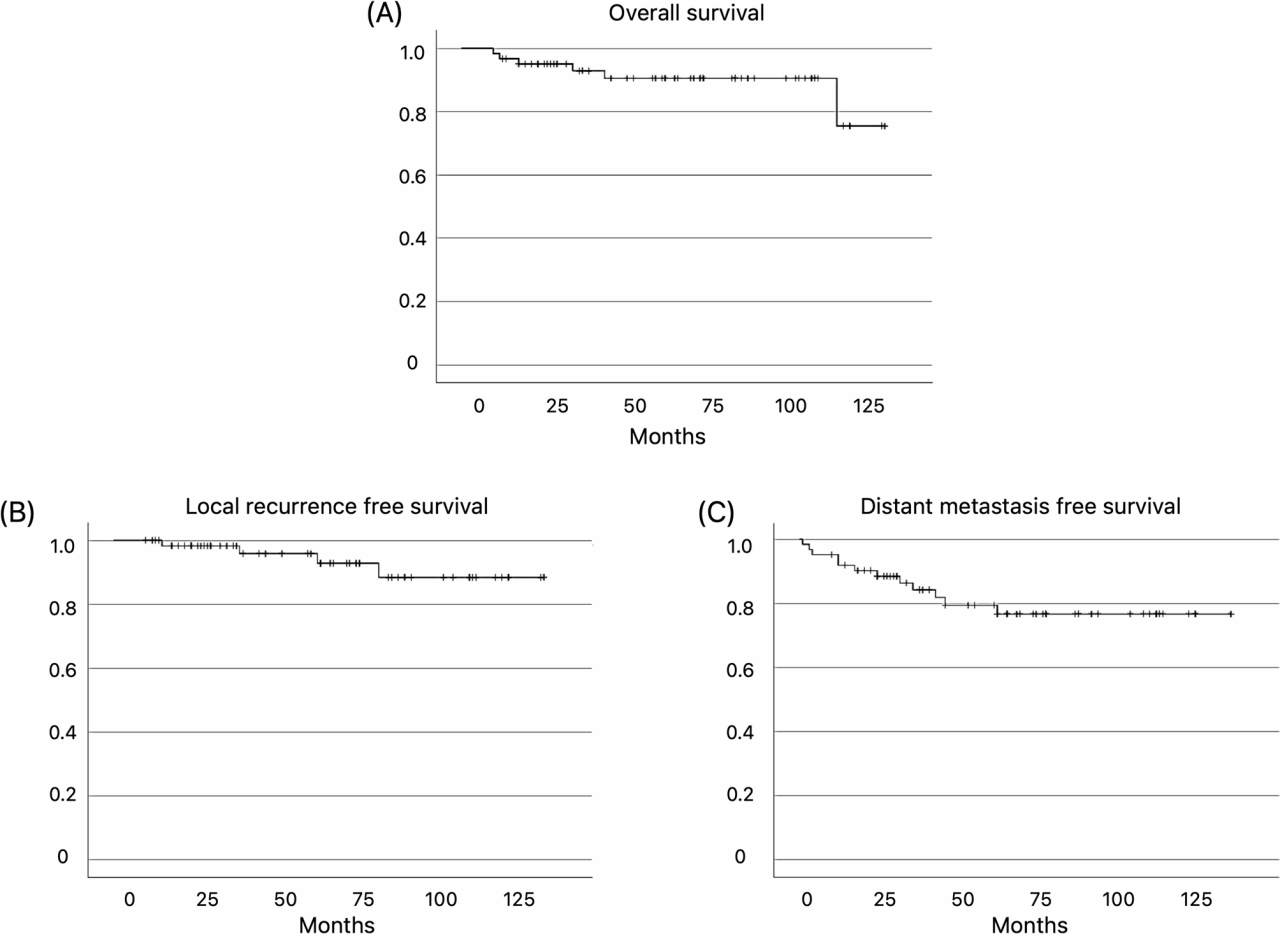
(**A**) Overall survival. (**B**) Local recurrence-free survival. (**C**) Distant metastasis-free survival

The 5-year overall survival, 5-year local recurrence-free, and 5-year distant metastasis-free survival rates were 90%, 96%, and 79%, respectively (Fig. 1). Knee joint function was measured at the last observation. The average knee flexion and extension angles were 127° and − 1.2°, respectively, with an average extension lag of 2.4°. The mean Lysholm score was 92.6 points.

Table 2 presents the associations between the Lysholm scores and various clinical factors. Factors significantly associated with lower Lysholm scores were female sex and histology such as MFS and UPS (*p* = 0.01 and 0.01, respectively).

**Table 2:** Association between the various clinical factors and the Lysholm score

Variables	Lysholm score (SD)	*p*
Age (year)		0.28
> 60	91.6 (10.5)	
< 60	93.5 (11.9)	
Sex		0.01
Male	96.7 (6.5)	
Female	89.4 (12.9)	
Tumor size (mm)		0.08
> 63	90.4 (12.3)	
< 63	95.0 (9.4)	
Histology		0.01
MFS, UPS*	86.3 (14.7)	
other	95.7 (7.2)	
Tumor grading		0.44
High grade	92.4 (11.1)	
Low grade	93.0 (12.1)	
Anterior or Posterior		0.45
Anterior	92.7 (10.5)	
Posterior	92.3 (12.0)	
Lateral or Medial		0.07
Lateral	95.6 (9.2)	
Medial	91.0 (9.8)	
Tumor Depth		0.19
Deep	93.8 (11.4)	
Superficial	90.9 (10.9)	
Surgical margin		0.19
R1	90.5 (12.0)	
R0	94.6 (11.1)	
Chemotherapy		0.49
Yes	92.6 (9.9)	
No	92.5 (12.0)	
Radiotherapy		0.06
Yes	83.0 (14.7)	
No	93.2 (10.8)	
Plastic reconstruction		0.32
Yes	91.8 (10.3)	
No	93.4 (12.4)	

* MFS: Myxofibrosarcoma, UPS: Undifferentiated pleomorphic sarcoma, SD: Standard deviation

**Fig. 2:**
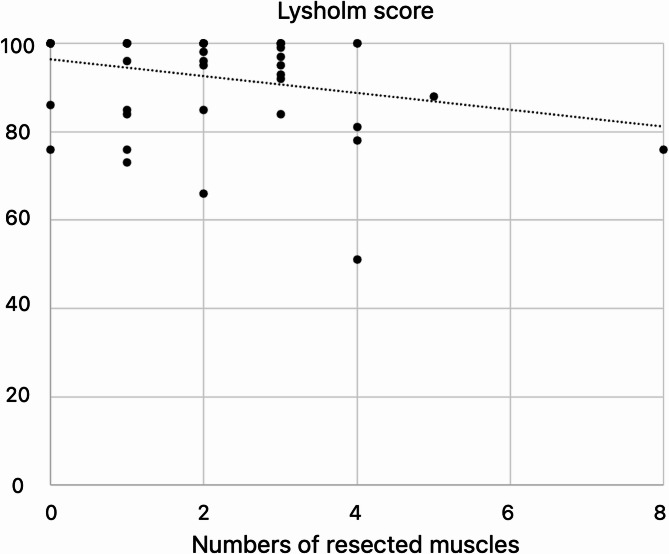
Correlation between the number of muscle resections and Lysholm score

**Fig. 3:**
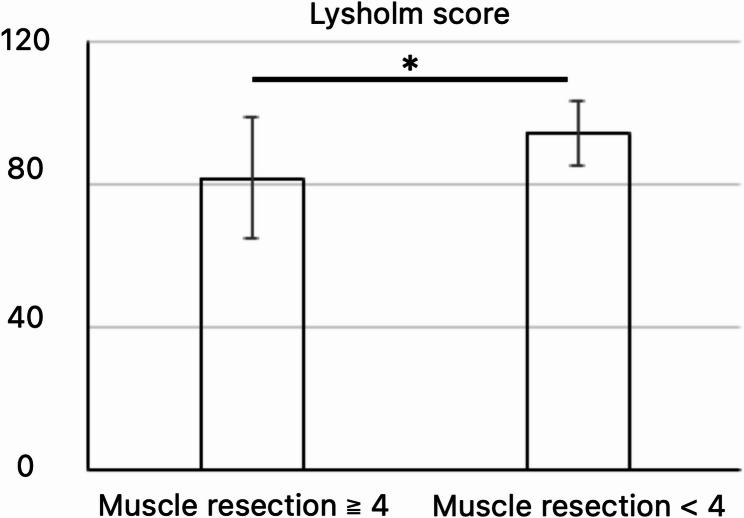
Comparison of Lysholm scores between patients with four or more muscle resections and those with fewer than four

There was a significant negative correlation between the number of resected muscles and Lysholm score (Fig. 2, *p* = 0.01, *r* = -0.331). Additionally, the group with ≥ 4 resected muscles had significantly lower Lysholm scores than the group with < 4 muscles resected (Fig. 3, *p* = 0.003); when comparing these groups, the former group had a significantly higher occurrence of deep-seated tumors (*p* = 0.01). The muscles more frequently resected in the group with ≥ 4 muscles included the adductor muscles, vastus medialis, medial hamstrings, rectus femoris, and triceps surae (Table 3).

**Table 3:** Comparison of various factors and types of resected muscles between groups with four or more muscle resections and those with fewer than four muscle resections

Variables	Muscle resection ≧ 4 (*n* = 7)	Muscle resection < 4 (*n* = 51)	*p*
Male sex (n)	4	20	0.366
Age (y, mean)	63	54	0.107
Tumor size (mm, mean)	103	61	0.081
Deep location (n)	7	25	0.011
High grade (n)	5	42	0.489
Hisgology (UPS, MFS, n)	2	19	0.654
Resected muscle			
Adductor (n)	3	1	< 0.001
Vastus medialis (n)	3	5	0.017
Sartorius (n)	3	8	0.086
Medial hamstrings (n)	4	9	0.019
Vastus lateralis (n)	2	5	0.153
Rectus femoris (n)	2	2	0.016
Triceps surae muscle (n)	4	4	< 0.001
Tibialis anterior (n)	0	2	0.594
Vastus intermedialis (n)	1	3	0.411
Biceps Femoris (n)	3	13	0.335

Table 4 shows the associations between the resection of specific tissues and Lysholm scores. Resections significantly associated with lower Lysholm scores included removal of the adductor muscles, patellar tendon, and medial collateral ligament (MCL) (*p* < 0.001, *p* = 0.002, *p* = 0.019, and *p* = 0.024, respectively).

**Table 4:** Association between Lysholm score and the resected tissues

Resected tissue	Lysholm score (SD)		*p*
	Resection (+)	Resection (-)	
Adductor muscles	74.2 (17.0)	94.2 (9.0)	< 0.001
Lateral collateral ligament	98.4 (2.3)	91.8 (11.6)	0.002
Patellar tendon	82.8 (14.5)	93.7 (10.3)	0.019
Medial collateral ligament	82.0 (14.1)	93.5 (10.5)	0.024
Sartorius	87.8 (15.4)	94.0 (9.3)	0.055
Vastus medialis	86.8 (12.2)	93.7 (10.7)	0.058
Medial hamstrings	89.0 (14.5)	93.9 (9.5)	0.091
Tibialis anterior	85.5 (13.4)	92.8 (11.1)	0.184
Triceps surae muscle	90.6 (10.2)	92.9 (11.4)	0.299
Vastus lateralis	94.3 (7.0)	92.2 (11.7)	0.341
Vastus intermedialis	91.3 (9.6)	92.6 (11.3)	0.424
Rectus femoris	93.5 (8.5)	92.4 (11.4)	0.431
Iliotibial tract	91.8 (14.5)	92.6 (10.9)	0.438
Biceps Femoris	93.1 (13.3)	92.2 (10.2)	0.829

*SD: Standard deviation

Table 5 summarize the results of the multiple regression analyses assessing the factors significantly associated with lower Lysholm score. Factors significantly associated with a lower Lysholm score included resection of the adductor muscles, female sex, histological subtype of MFS or UPS, and the group with ≥ 4 resected muscles. (*p* < 0.026, *p* = 0.006, *p* < 0.001, and *p* = 0.024, respectively). Adductor muscle resection group had a significantly higher number of muscles resected during wide tumor resection (*p* < 0.001), and tended to have larger tumors (*p* = 0.06).

**Table 5:** Multiple regression analysis of factors related to Lysholm scores

Variables	Odds Ratio	95% CI (Lower– OR)	95% CI (Upper– OR)	*p*
Adductor muscles	-2.353	-17.836	-1.355	0.026
Lateral collateral ligament	1.171	-3.083	11.574	0.249
Medial collateral ligament	-1.632	-14.870	1.583	0.110
Male sex	2.875	1.949	11.169	0.006
Histology (MFS, UPS*)	-3.860	-13.876	-4.339	< 0.001
More than 4 muscles resected	-2.353	-17.836	-1.355	0.024

* MFS: Myxofibrosarcoma, UPS: Undifferentiated pleomorphic sarcoma

Details the nine cases in which either the MCL or lateral collateral ligament (LCL) was resected. Following ligament resection, four patients underwent ligament reconstruction using autograft transplantation. In three cases, LCL reconstruction was performed. In each case, transverse bone tunnels were created in the femur and anterior–posterior tunnels in the fibula, autologous semitendinosus and gracilis tendon grafts were harvested, and fixation was achieved using RCI screws. MCL reconstruction was carried out in a similar fashion, employing semitendinosus and gracilis tendon grafts. The group that underwent reconstructive procedures showed significantly better results in the limp category (*p* = 0.04) of the Lysholm score and a trend in favor of the instability category of the Lysholm score and total score (*p* = 0.06 and 0.06, respectively)

**Table 6:** Clinical characteristics of 9 cases with combined resection of the MCL or LCL.

Case	Age (years)	Sex	Tumor size (mm)	Histology	Resected ligament	Plastic reconstruction	Follow up (Months)	Knee flexion	Knee extension	Extension lag	Ligament reconstruction	Lysholm score
1	14	F	70	Synovial sarcoma	LCL	GC	132	130	0	0	Yes	97
2	43	F	68	Myxofibrosarcoma	LCL	LD	111	95	0	10	Yes	95
3	71	F	8	Myxofibrosarcoma	MCL	GC	73	120	0	60	No	66
4	58	M	97	CIC-rearranged sarcoma	LCL	GC	20	130	0	0	Yes	100
5	47	M	22	Leiomyosarcoma	MCL	ALT	75	130	0	0	Yes	100
6	17	F	25	Synovial sarcoma	LCL	No	105	130	0	0	No	100
7	80	M	20	UPS	LCL	GC	60	130	0	0	No	100
8	65	F	50	UPS	MCL	GC	64	110	0	0	No	84
9	56	M	133	Myxoid liposarcoma	MCL	LD	22	100	-10	10	No	78

M: Male, F: Female, UPS: Undifferentiated pleomorphic sarcoma, GC: Gastrocnemius, LD: Latissimus dorsi, ALT: Anterolateral thigh

## Discussion

This study is the first to evaluate and report the functional prognosis and factors of sarcomas around the knee joint using the Lysholm score based on clinical outcomes from eight representative institutions in the Tokai region of Japan that primarily treat bone and soft tissue tumors. We uniquely incorporated the Lysholm score, which is specifically tailored to evaluate function in cases of ligament injury around the knee joint. Furthermore, we evaluated the factors influencing functional scores, including resected tissues, with a focus on ligament reconstruction. These aspects distinguish our study from previous reports and are noteworthy features thereof.

Clinical factors such as sex and specific tumor histology were associated with functional scores. Considering the association between sex and functional decline, women generally have lower muscle mass, lower muscle strength, and smaller skeletal structures than men. This may have led to insufficient compensation for residual function after extensive tumor resection or a less effective recovery through rehabilitation. Additionally, the effect of sex on patient-reported outcomes has been documented in previous studies [10], supporting the validity of our findings. Some MFS and UPS exhibit strong infiltration into surrounding tissues, with a wide spread over the fascia known as the “tail sign” [11], a common finding. The resection of such tumors typically requires setting resection margins that include the tail sign, leading to larger resections and defective areas. Additionally, the likelihood of positive surgical margins and the subsequent need for postoperative radiotherapy increases. Although we have not individually reviewed whether these cases exhibited the tail sign, it is plausible that these factors contributed to the functional decline.

Perioperative RT has the potential to increase the local control rate but may carry a risk of postoperative infection [12]. In this cohort, 5 patients received RT and one of them developed wound complication. Of the 58 patients who did not receive RT, 6 developed wound problems. In this study, there were few cases of RT and few cases of infection, making it difficult to demonstrate a statistically significant difference.

In this study, muscle flaps were performed in 35 cases: pedicled gastrocnemius flaps in 21, free latissimus dorsi flaps in 8, and anterolateral thigh flaps in 6. There was no significant difference in Lysholm scores between patients who did and did not undergo muscle flap reconstruction. In the cases requiring muscle flaps, defect coverage and flap suturing that supported ligament reconstruction may have contributed to preserved knee function. Conversely, patients who did not require flaps likely had smaller defects, resulting in fewer functional impairments. Additionally, variations in flap technique (pedicled versus free) may affect operative time and postoperative immobilization, potentially influencing functional outcomes; these factors warrant investigation in future studies.

In the pathological evaluation of the margins after wide resection in this study, the margins were well maintained, with R1 in 4 cases and R0 in all other cases. However, if the tumor is very close to the ligament or tendon, if the surgeon is prepared to perform ligament reconstruction, and if the techniques and methods are mature, it is possible to perform a more robust surgical approach to cure without reducing the margin of resection. From this perspective, we believe it is important not to hesitate to fully consider and prepare for ligament reconstruction.

In our cohort, 35 sarcomas arose in the deep compartment and 28 in the superficial compartment, with no significant difference in postoperative function between the two groups. Even in superficially located tumors, an average of one muscle or ligament resection was required. The anatomical region surrounding the knee joint is characterized by a high density of soft tissue structures within a confined space; therefore, superficially arising sarcomas that abut the fascia or collateral ligaments often require deep-margin excision that includes these tissues.

In this study, adductor muscle, patellar tendon, and MCL were significantly associated with lower Lysholm scores in univariate analysis and adductor muscle resection was significantly associated with lower Lysholm scores in multivariate analysis. Four or more muscle resections were also significantly associated with lower Lysholm scores. Resection of the adductor muscles, which are large, deep-seated muscles attached to the pelvis and femur, plays a crucial role in stabilizing the pelvis and is significantly associated with lower Lysholm scores. The proximity of the adductor canal and the convergence of muscle groups near the knee joint necessitates the resection of many muscles when tumors were involved, impacting nerve and vascular dissection, and contributing to postoperative lymphedema. The tumor requiring concomitant resection of the adductor muscle appears to be located medially and slightly posterior to the distal femur, affecting the functions that contribute to knee joint flexion and femoral adduction. These contribute not only to movement during gait but also to stability of the lower limb and may be the cause of the effect on lower limb function, including gait. Tanaka et al. reported that while resection of a single adductor muscle does not significantly impair function, the removal of multiple adductor muscles results in significant functional decline [13].

Several previous reports have investigated the resection of lower extremity STS and its impact on function and quality of life (QOL). Tanaka et al. reported 17 cases of STS originating in the lower limbs, focusing on the decline in flexor muscle strength, lower limb functional impairment, and decreased QOL following concurrent resection of the flexors [14]. They concluded that a reduction in flexor strength significantly affected the lower limb function and QOL. Similarly, they investigated 18 cases of STSs originating in the thigh and examined the effects of quadriceps muscle resection on muscle strength, functional impairment, and QOL [15]. Their findings indicated that increased resection of the quadriceps muscles significantly exacerbated lower limb functional impairment and decreased QOL. Although the tumor locations in our study differed from theirs, our research is similar in that it investigated the association between soft tissue resection and limb function. In our study, univariate analysis showed a significant correlation between the number of resected muscles and Lysholm score, yielding similar results. Their work differs from ours in that they conducted detailed evaluations of muscle strength using specialized equipment and assessed QOL. In contrast, our study analyzed and examined various factors that might be associated with functional impairment, such as radiation therapy, muscle flap procedures, ligament reconstruction, and the number and type of tissue resections. Here, we have detailed our findings regarding ligament reconstruction. Fukushima et al. reported 90 cases of STSs and discussed the association between early postoperative physical function and lower limb functional impairment [16]. They assessed pre- and postoperative Timed Up and Go tests (TUGT) and MSTS/ISOLS scores and found correlations between pre- and postoperative results. Resection of the quadriceps and tibialis anterior muscles was related to differences in the TUGT scores before and after surgery. The authors concluded that identifying the resected muscles is crucial for early postoperative rehabilitation. Our research also identified significant functional impairments related to the resection of specific tissues such as the adductor muscles, and MCL. In postoperative rehabilitation, it is essential to closely share the details of the surgery with physical therapists, including the type and number of tissues resected, ligament reconstruction, and the presence and methods of plastic surgical reconstruction. Discussing the optimal methods and schedules for physical therapy can significantly contribute to functional recovery.

Although multivariate analysis did not show significant differences, we paid special attention to nine cases that involved resection of either the MCL or LCL. Among these patients, four underwent ligament reconstruction surgery. Patients with ligament reconstruction exhibited significantly better outcomes in the Limp category and performed better on most other items of the Lysholm score than those without reconstruction.

Among the five cases in which the LCL was resected, reconstruction was performed in three cases. Numerous reports exist in the trauma literature. LCL injuries are classified as type 1 to type 3 based on the severity of damage [17–20]. Surgical indications included grade 3 or complex injuries involving other tendinous components [21]. Wide resection of STS often involves defects greater than complete rupture and is frequently accompanied by substantial loss of surrounding soft tissues, thus fitting the criteria for reconstruction, as described. In our series, LCL reconstruction was performed using a nearly uniform technique in all cases, resulting in excellent postoperative functional preservation. Although our sample size precludes a detailed comparison of different reconstruction methods, cases in which transverse femoral and fibular head bone tunnels were created and autologous or synthetic grafts were used demonstrated particularly favorable functional recovery. Standardizing the reconstruction technique may facilitate procedural proficiency and further optimize knee joint function.

Various reconstruction methods have been reported [17]. Clancy et al. described a technique for transposing the biceps femoris tendon to the anterior iliotibial tract [22]. Latimer et al. reported the use of a patellar tendon allograft [23], while methods utilizing the Achilles tendon [24, 25] and semitendinosus have also been documented. Although various studies have been conducted, the most effective method remains controversial. Additionally, the condition after STS resection varies among individuals, making it difficult to standardize the approach as in trauma cases. After LCL resection for STS, it is necessary to consider these methods and assess specific post-resection conditions to determine the most appropriate reconstruction method for the patient’s knee. Whether the reconstruction is anatomical, the degree of knee flexion during fixation and the tension during reconstruction are critically important surgical techniques.

Among the four cases requiring MCL resection, reconstruction was performed in one case using the semitendinosus and gracilis tendons. Similar to LCL cases, complex injuries involving the MCL can lead to valgus and rotational instability [26]. Following the resection of STS, defects comparable to those observed in complex injuries are presumed, making ligament reconstruction desirable. Various reconstruction methods have been reported [27], all of which have achieved favorable outcomes. The widely used semitendinosus tendon technique involves the creation of bone tunnels and passing the tendon through the lateral aspects of the femur and tibia for fixation. In this study, MCL reconstruction was also performed using the ST tendon, resulting in excellent outcomes, with a Lysholm score of 100.

There are various reports on the patellar tendon reconstruction in trauma. Reconstruction is mainly performed using autografts of the semitendinosus and gracilis tendon [28]. Reconstruction is frequently reported in the field of tumors, particularly in cases of malignant bone tumors in the proximal tibia [29]. In this study, one patient underwent combined reconstruction of the LCL and the patellar tendon, while another underwent isolated patellar tendon reconstruction. In the combined case, a patellar bone tunnel was drilled and an artificial ligament, augmented with an autologous semitendinosus tendon graft, was fixed from the quadriceps tendon to the tibia. In the isolated case, reconstruction was performed using an iliotibial band graft. Only one patient underwent multiple ligament reconstructions, and this individual demonstrated excellent knee function, achieving a Lysholm score of 95. All patellar tendon resection cases were treated with muscle flaps. For patellar tendon reconstruction, not only ligament reconstruction, but also functional support with muscle flaps and the involvement of remaining tissues such as the ITT and tibialis anterior muscle are important factors. In this study, the number of cases of patellar tendon reconstruction was small, and detailed evaluations to discuss the necessity of reconstruction were not conducted. Further investigations are required for these evaluations. The patellar tendon is responsible for kicking and stepping when walking. Impairment of knee extensor function, including the patellar tendon, may have had a significant impact on loss of knee joint function.

The decision of whether to perform ligament reconstruction is very important. In patients with large tumors or those who require multiple soft tissue resections due to extensive contrast enhancement, knee instability after wide resection of the tumor may be severe and ligament reconstruction may show a suggestive benefit for improving the knee function. In cases of lateral knee tumor resection, we have seen a high degree of instability in patients who undergo concomitant resection of the ITT in addition to the LCL, whereas the remaining ITT seems to be less unstable. This may explain why it is more prone to instability than the LCL. Evaluation of instability after ligament resection is crucial, and robust reconstruction is recommended in cases of significant instability. The indications for ligament reconstruction may vary based on individual activities of daily living (ADL) demands. Optimal limb function can be achieved by reliable tumor resection, robust fixation, precise ligament balancing, and appropriate postoperative care. However, the increased surgical time and risk of infection associated with the use of prosthetics, such as screws or staples, for ligament fixation require careful consideration before proceeding with ligament reconstruction. Although conservative treatment is often adequate for isolated MCL or LCL injuries, complex injuries involving medial or posterolateral corners and instability require surgical reconstruction [30]. In addition, the potential development of secondary osteoarthritis after ligament resection must be considered [31]. Surgeons should consider the appropriateness of ligament reconstruction based on these long-term outcomes. It is vital to assess each case of periarticular STS resection to determine its suitability for ligament reconstruction.

Rehabilitation following wide tumor resection and soft tissue reconstruction is crucial for optimizing knee and gait function. In most of our ligament-reconstructed cases, patients underwent several weeks of splint immobilization with non–weight-bearing, followed by conversion to a knee orthosis and gradual initiation of weight-bearing and range-of-motion exercises. After collateral ligament reconstruction, protected ambulation in an orthosis is recommended to safeguard graft integrity, with rehabilitation protocols developed in collaboration with trauma and knee specialists.

Long-term vigilance for graft laxity and the development of secondary osteoarthritis is essential. Risk factors for osteoarthritic change include patient age at surgery, preexisting joint degeneration, extent of soft tissue resection, and intraoperative ligament balance. Even after completion of oncological follow-up, annual radiographic evaluation of the knee is advised to monitor for degenerative changes.

This study has several limitations. This study is a small cohort of 63 cases and is a retrospective study. In the subgroup analysis of ligament reconstruction, the statistical power is low because there were 4 reconstructed cases. The borderline significance of ligament reconstruction (*p* = 0.06) should be considered exploratory, and a large prospective study is recommended for validation. In addition, the cohort in this study was limited to Japanese. We believe that future multinational studies in more centers are needed to increase the generalizability by ethnicity.

This retrospective study acknowledges the variability in treatment approaches across different facilities, including variations in adjuvant or neoadjuvant radiotherapy and chemotherapy. Although surgical excision with adequately wide margins is standard, the extent of margins and indications for reconstructive and functional surgery vary slightly between institutions. Postoperative limb immobilization and rehabilitation programs are not standardized, which may influence outcomes. Despite these variations, the Tokai Musculoskeletal Oncology Consortium holds conferences to discuss treatment methodologies, ensuring a consistent treatment philosophy and a sufficient level of care across all participating facilities. All institutions involved are specialized bone and soft tissue tumor hospitals that provide the highest level of treatment. Despite some variations, this cohort maintained consistent treatment levels.

This study evaluated the presence and absence of soft tissue resection in a binary manner. However, in actual surgeries, the amount of tissue resected and whether continuity is preserved vary. Evaluating these resection details is extremely difficult and was not performed in the present study.

This study used the Lysholm score, which is specific to the assessment of knee joint function after knee ligament resection and is an appropriate functional assessment index, but its scope is narrow. Broader quality of life measures such as the TESS, SF-36, and EORTC QLQ-C30 are functional assessment measures that have received much attention in recent years, and supplementing these measures in future studies would increase their comprehensiveness. The minimally clinically important difference of the Lysholm score was reported to be 11 [32]. Although the significance of the Lysholm score in this study was adductor muscle resection, sex, histological type, and number of muscular resections, only a few factors had Lysholm score difference greater than 11, which we consider to be another limitation of this study.

## Conclusions

This study investigated functional outcomes following resection of soft tissue sarcomas around the knee joint. Lower Lysholm scores were significantly associated with resection of the adductor muscles, female sex, histological types such as MFS and UPS, and the group with ≥ 4 resected muscles. The results of this study suggest that for cases where the MCL or LCL were resected, thorough evaluation and consideration of the necessity of ligament reconstruction are required, as ligament reconstruction may give a suggestive benefit for improving the function in cases requiring resection of the LCL or MCL.

## Data Availability

The data that support the findings of this study are not openly and are available from the corresponding author upon reasonable request. Data are located in controlled access data storage at Hiroshi Koike.

## Abbreviations

STS: Soft tissue sarcoma,
UPS: Undifferentiated pleomorphic sarcoma,
MFS: Myxofibrosarcoma
MPNST: Malignant peripheral nerve sheath tumor,
MCL: Medial collateral ligament,
LCL: Lateral collateral ligament,
QOL: Quality of life,
TUGT: Timed up and go tests,
ADL: Activities of daily living
